# Fluorescent Probe for Ag^+^ Detection Using SYBR GREEN I and C-C Mismatch

**DOI:** 10.3390/bios11010006

**Published:** 2020-12-24

**Authors:** Xiaohong Zhou, Abdul Ghaffar Memon, Weiming Sun, Fang Fang, Jinsong Guo

**Affiliations:** 1 Center for Sensor Technology of Environment and Health, State Key Joint Laboratory of ESPC, School of Environment, Tsinghua University, Beijing 100084, China; 18095131210068@hainanu.edu.cn; 2 Department of Environmental Engineering, NED University of Engineering and Technology, Karachi 75270, Pakistan; abdulghaffar@neduet.edu.pk; 3 Key Laboratory of the Three Gorges Reservoir Region’s Eco-Environments of MOE, Chongqing University, Chongqing 400030, China; fangfangcq@cqu.edu.cn

**Keywords:** silver ion detection, C-C mismatch, SYBR Green I, fluorescent probe

## Abstract

Among heavy metals silver ions (Ag^+^) severely impact water, the environment and have serious side effects on human health. This article proposes a facile and ultrasensitive fluorescent probe for the detection of Ag^+^ ions using SYBR Green I (SGI) and cytosine-rich (C-rich) silver-specific oligonucleotide (SSO). Maximum fluorescent intensities with the highest sensitivity were obtained using a 0.61 dye/SSO base ratio (DBR). The established sensing principle using the optimized parameters for bath temperature, SSO concentration, DBR, ionic strength, pH, reaction time, incubation duration and temperature effect achieved a sensitive limit of detection of 59.9 nM for silver ions (calculated through 3σ, n = 11) with a linear working range of 100–1000 nM and 0.997 R^2^. The total time for one assay is below 10 min; The relative standard derivation for ten repeated measurements is 8.6%. No blatant interferences were observed in the selectivity test when fluorescent probe is evaluated by investigating the effects of 11 common interference factors in the aqueous matrix. In extreme cases, three false-negative factors were observed, including calcium hardness, magnesium hardness, and hypochlorite. The recovery ratios were within the range of 79~110% for three types of diluted water.

## 1. Introduction

Among the heavy metal ions silver ions (Ag^+^) are widely used due to their unique antimicrobial properties which are toxic and cidal to various bacterial, viral, algal and fungal species and are extensively applied in medicines, cosmetics and building materials [1,2]. However, excessive uptake has a range of severe health consequences, including inhibition of growth and development, cancer, organ damage, nervous system damage, cytotoxicity, mitochondrial dysfunction, and death in extreme cases [2,3,4,5,6]. Human exposure to heavy metal ions derives from natural waters polluted by industrial sources, including coal and gold mining, solid waste incineration, wood pulping, combustion of fossil fuel, and chemical processing [7]. Owing to the hazardous impact of silver ions, the United States Environmental Protection Agency (USEPA) has regulated the maximum permissible level of Ag^+^ in drinking water to approximately 900 nM [8]. In order to protect the environment and human health, heavy metal detection is ubiquitous and frequent not only for contaminant warning but also for trace metal quantification in drinking water sources, public water supplies, groundwater, etc. Currently, the widely used quantification of heavy metal has been limited to traditional analytical methods, including inductively coupled plasma mass spectrometry, atomic absorption spectroscopy, and atomic fluorescence spectrometry [9]. Although precise and constrained in detection, these methods are labor-intensive, require costly and sophisticated instrumentation, as well as complicated and multistep sample preparation, which preclude online and real-time monitoring. Consequently, many initiatives have been taken to detect heavy metals cost-effectively, rapidly, and in the field through colorimetric, optical, and electrochemical sensors [7,10,11,12].

Very recently, oligonucleotide-based probes have been designed for heavy metal ions analysis and even for the multiplexed analysis of two metal ions. The probes are commonly designed using two kinds of functional nucleic acid. One is based on metal ion-dependent DNAzymes, which can irreversibly cleave DNA after the introduction of metal ions due to a metal ion-dependent catalytic reaction. DNAzymes have been successfully designed to detect Cu(II), Pb(II), Hg(II), UO_2_(II), and K(I) with high selectivity [11,13,14,15,16]. Among them, fluorescent DNAzyme-based sensors have attracted increasing attention owing to their high sensitivity and selectivity. Part of the sensor has realized commercialization (http://www.andalyze.com/). The detection limits are below to nM level and even pM for UO_2_(II) analysis [17]. However, DNAzyme-based fluorescent sensors usually require two or more quenchers labeled at one DNA strand in order to reduce the background [18], which makes them expensive and labor-intensive. Therefore, searching for the label-free or less-labeled response strategy is a promising research direction. The other is based on heavy metal ions’ ability to selectively bind to base-base (thymine-thymine or cytosine-cytosine) mismatched pairs in DNA to form complexes. It has recently been proven that oligonucleotides would be a useful tool for Ag^+^ detection via the formation of an Ag+-medicated base pair, cytosine-Ag^+^-cytosine (C-Ag+-C) [19]. Similarly, Hg^2+^ can specifically interact with the thymine-thymine (T-T) mismatch in DNA duplexes to form the T-Hg^2+^-T complex [10]. Ag^+^ and Hg^2+^ sensors based on fluorescence or electrochemistry using DNA duplexes or hairpin construction have been developed [20].

SYBR GREEN I (SGI) is a fluorescent double-stranded DNA (dsDNA) stain often used in molecular biology applications. Since its introduction in the early 1990s, the monomeric cyanine dye, SGI, has been used to test nucleic acids. SGI has a high affinity for dual-stranded DNA (dsDNA). Its fluorescence increased dramatically when bound to dsDNA, at least 11-fold higher than ssDNA binding. Besides, the fluorescence signal was not significantly influenced by the assay conditions (pH, length, and time). These benefits make it ideal for measuring low concentrations of ssDNA samples or DNA amplification materials, even in the presence of other nucleic acids, such as RNA, ssDNA, and nucleotides [21]. The unique preference of SGI towards dsDNA enable it an excellent candidate to develop the label-free technology for the detection of heavy metals.

Therefore, the goal of this work is to develop an easy and sensitive method for the detection of Ag^+^ by using SGI and Ag^+^-specific cytosine-rich oligonucleotides. We chose silver ions as the targets because they act as one of the more severe environmental pollutants and have serious side effects on human health. Under the systematic verification and optimization of eight detection conditions, the sensitivity, selectivity, repeatability and practicability of this method have been thoroughly evaluated. Besides, this is the first work to systematically investigate the effects of the major aqueous matrix, including 11 common interference factors, on the detection performance. These findings can provide further insight into the realistic implementation of this approach.

## 2. Materials and Methods

### 2.1. Material

The Ag^+^-specific cytosine-rich oligonucleotides (5′-CTC TCT TCT CTT CAT TTT TCA ACA CAA CAC AC-3′, SSO) [19] were purchased from Shanghai Sangon Biotechnology. Co., Ltd. (Shanghai, China). One package of SSO powder was centrifuged at 12,000 rpm for 2 min. Then, 35 μL DI water was added into the centrifuge tube and well mixed to achieve the final concentration of 100 μM SSO as the stock solution. The stock solution was stored in a refrigerator at 4 °C. A fixed concentration of SSO solution was freshly prepared by diluting the stock with DI water for use.

SGI (10,000×) was purchased from Beijing Solarbio Science & Technology Co., Ltd. (Beijing, China) and diluted by using DMSO to a final concentration of 100× as the stock solution. The stock solution was stored in a refrigerator at 4 °C. The predefined concentration of SGI was freshly prepared by diluting the stock with DMSO for use. 1000 μg/mL single element standard solution of mercury in 1.5 mol/L HNO_3_, AgNO_3_, and other metal salts were analytical reagent grade and purchased from Sinopharm Chemical Reagent Co., Ltd. (Beijing, China). DI water was used throughout the experiments. Fluorescent emission spectra were recorded on a F-7000 fluorescence spectrometer (Hitachi, Tokyo, Japan).

### 2.2. Feasibility of Ag^+^ Detection by Using SSO and SGI

NaNO_3_ (200 mM) and appropriate concentration of Ag^+^ (0, 100, 200, 300, 500, 700 nM) were added separately into HEPES buffer (10 mM, pH 7.1), followed by the addition of SSO (10 nM), reacted for 20 min in the dark (37 °C) to form the C-Ag^+^-C complex at room temperature. Then SGI was added into the mixture to the final 0.25× and incubated at room temperature for 5 min before the fluorescence was measured. Fluorescence (FL) intensity was recorded at 530 nm with an excitation wavelength of 497 nm.

### 2.3. Optimization of Ag^+^ Detection Conditions

The effects of the SSO concentration, SGI/SSO ratio, salt concentration (NaNO_3_), pH value, reaction time, and temperature on the Ag^+^ detection performance were investigated using the procedures stepped as follows.

A 10 μL SSO, 80 μL Ag^+^, and 10 μL 100 mM MOPS buffer solution was mixed and reacted for 10 min in the dark to form the C-Ag^+^-C complex. Then 5 μL SGI was added into the mixture and incubated in the dark for another 2 min. Moreover, the reaction time and the incubation time remained 10 min and 2 min, respectively, if not specified. Finally, 695 μL DI water was added to the final volume of 800 μL before the fluorescence was measured. All bioassays were kept at room temperature except in the temperature optimization experiments. FL intensity was recorded at 530 nm and 500 nm with an excitation wavelength of 497 nm. It should be noted that the concentrations mentioned below donate the original values before the mixture.

The relative FL intensity, i.e., ratios of fluorescence intensities with and without Ag^+^ as the optimization criteria were applied. The higher the relative fluorescence intensity is, the lower the signal-to-noise ratio is. The differences in the value of FL intensity with and without Ag^+^ determined the sensitivity of the method.

### 2.4. Selectivity

The impact of 11 to 15 interference factors, including mono–, di–, and trivalent cations, metal ions, anions, and hardness commonly existed in the aqueous matrix, were subjected to the selectivity assay. Concentrations of the interference ions were spiked as amalgam containing a mixture. The tolerance level for calcium and magnesium hardness by diluting the interference ions was also investigated in the assay.

### 2.5. Recovery

Three types of real water matrix were chosen for the recovery experiment, including two types of surface water (Danjiangkou Reservoir and Hetang Lake on the Tsinghua campus) and one groundwater type. The Danjiangkou Reservoir is the water source for the middle route of the South-to-North Water Diversion Project in China. Thus, its water quality status is vital. Hetang lake is on the Tsinghua campus, which is a small lake without input and output. The groundwater is taken from hundreds of meters underneath the Tsinghua Yuan with the hardness of 290–350 mg/L in calcium carbonate.

## 3. Results and Discussion

### 3.1. Sensing Mechanism

**Figure 1:**
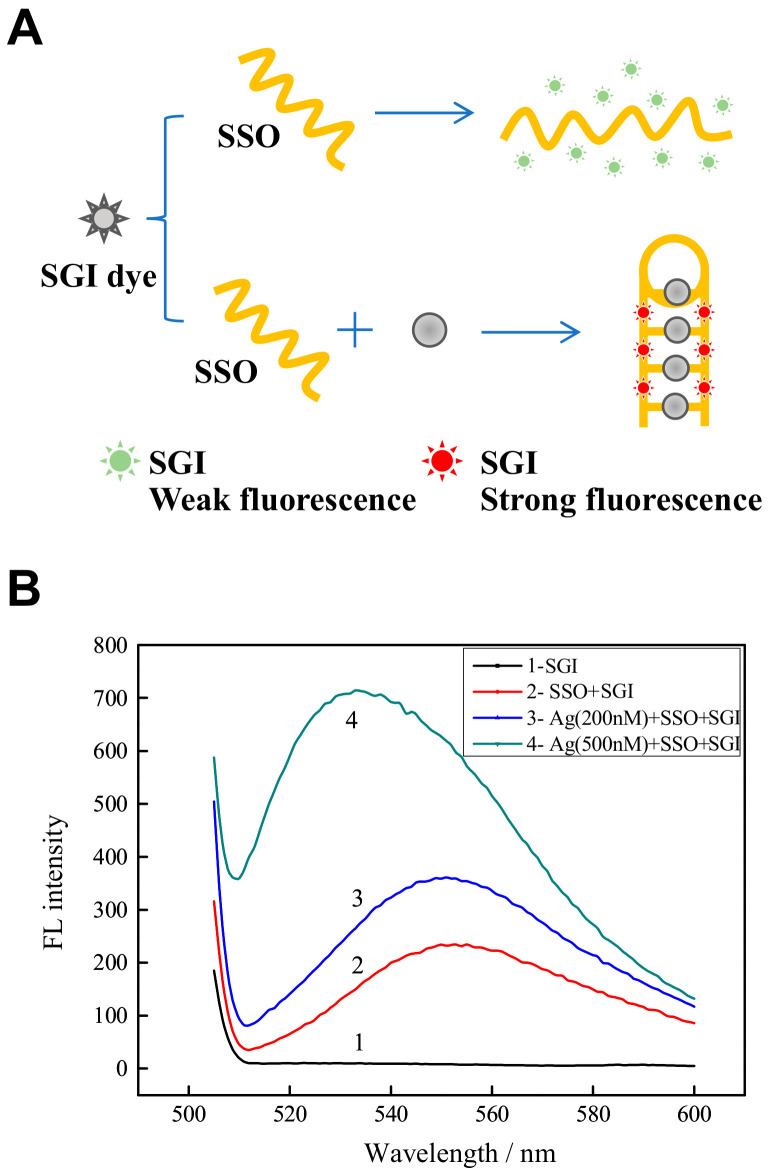
Schematic diagram of using Ag^+^-specific C-rich oligonucleotides SSO and SGI for Ag^+^ detection: (**A**) Schematic representation of the “turn-on” fluorescence detection of Ag^+^; and (**B**) Fluorescence spectra caused by the SSO and SGI system at different conditions. Curve 1-4 represent 1× SGI, 1 μM SSO + 1× SGI, 1 μM SSO + 1× SGI + 200 nM Ag^+^, 1 μM SSO + 1× SGI + 500 nM Ag^+^, respectively, at pH = 7.0.

Figure 1A represents the schematic diagram of using Ag^+^-specific C-rich oligonucleotides and SGI for silver detection. Briefly speaking, SSO retains their arbitrarily coiled form in the absence of Ag^+^. The fluorescence of such a complex SSO and SGI (SGI/SSO) was very low because the interaction between randomly coiled SSO and SGI is weak [21]. The presence of Ag^+^, in contrast, leads to the formation of C-Ag^+^-C base pairs [19], enabling SSO from an unusually coiled structure to a hairpin-like structure to shift its shape. Since the hairpin structure of SGI has a high affinity, there is a heavy fluorescence of the SGI/SSO complex. Consequently, this “turn-on” fluorescence process allows Ag^+^ to be recognized.

### 3.2. Feasibility of This Method for Ag^+^ Detection

The feasibility of this method for Ag^+^ detection has been validated, as shown in Figure 1B, which displays how the SGI fluorescence shifted in varying environments. Initially, SGI’s aqueous solution in 10 mM HEPES buffer displayed virtually no fluorescence (curve 1). The SGI/SSO complex exhibited very low fluorescence (curve 2), demonstrating that the randomly coiled SSO had some secondary structures by hydrogen bonding and weakly interacted with SGI. This phenomenon accorded well with the previous results reported by other researches [19,22]. However, with the addition of 200 nM and 500 nM Ag^+^, the fluorescence of the SGI/SSO complex increased significantly (curve 3, 4), thus increasing the fluorescence signal of mixture. This finding showed the SSO had folded into a C-Ag^+^-C hairpin-like structure, interacting strongly with SGI. These results suggest that the method described here could potentially be applicable to Ag^+^ detection.

### 3.3. Optimization of Experimental Conditions for Ag^+^ Detection

#### 3.3.1. Effect of Bath Temperature for SSO

**Figure 2:**
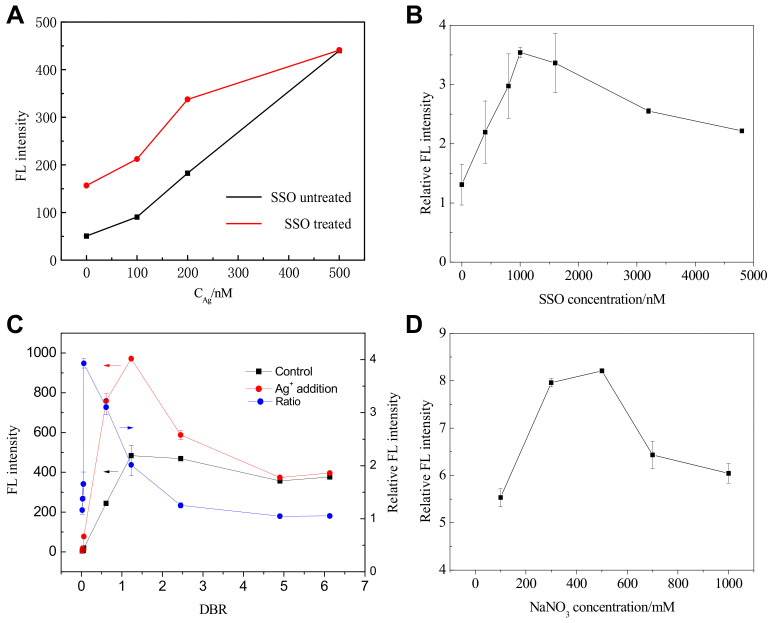
Effects of (**A**) bath temperature for SSO; (**B**) SSO concentrations; (**C**) dye/SSO base ratio, i.e., DBR value; and (**D**) ionic strength on fluorescence intensity for Ag^+^ detection (n = 2 if not specified).

The low background fluorescent value of SGI depends on the proper structural orientation of SSO as other dsDNA stains [21,23]. It is well known that the bath temperature would affect the secondary structure of DNA sequence [24], hence affecting the background fluorescence. The effect of SSO’s bath temperature on the fluorescence response of SGI at different conditions was examined, and the results are shown in Figure 2A. When the SSO was heated to 88 °C for 10 min, the background fluorescence value of SGI-SSO was higher than SSO untreated, indicating that the pretreatment of heating has an adverse effect on the elimination of background value. Considering the lower background value and detection limit, we chose the SSO unheated for the metal ions detection throughout the experiments.

#### 3.3.2. Effect of SSO Concentration and Dye/SSO Base Ratio for Ag^+^ Detection

It is well known that the SGI binds to dsDNA through both intercalation and minor groove binding as other dsDNA stains, which should be related to the variable SSO concentrations and SGI concentrations [19,21,22,23]. Therefore, the effect of variable SSO concentrations on the Ag^+^ detection performance was firstly investigated under the fixed conditions in the measurement procedure of C_Ag_ = 10 μM (to a final concentration of 1 μM), SGI = 1×, 100 mM MOPS buffer solution containing 500 mM NaNO_3,_ and pH = 7.0. Moreover, the parallel experiments without Ag^+^ addition were conducted for the control. Ratios of fluorescence signals with and without Ag^+^ (Relative FL intensity, %) were recorded for comparison and shown in Figure 2B. When 1 μM SSO concentration was used (final concentration of 12.5 nM), the relative FL intensity reached the most significant value of 3.54 ± 0.09, indicating the lowest signal-to-noise ratio. The decreased relative FL intensity at high SSO concentration might be attributed to the SSO intermolecular mismatch caused by Ag^+^. Therefore, a 1 μM SSO concentration was chosen in the following experiments.

Subsequently, the effect of dye/SSO base ratio (DBR) on the Ag^+^ detection performance was investigated by varying the SGI concentrations under the fixed conditions of C_Ag_ = 10 μM, SSO = 1 μM, 100 mM MOPS buffer solution containing 500 mM NaNO_3,_ and pH = 7.0. Moreover, parallel experiments without Ag^+^ addition were conducted for the control. The Ag^+^-specific oligonucleotide probe consists of 32 bases. It is also known that the molar concentration for SGI 1× is equal to be 1.96 μM. Therefore, the investigated DBRs are 0.02, 0.03, 0.05, 0.06, 0.61, 1.23, 2.45, 4.9 and 6.13, respectively, which correspond to the SGI 0.25×, SGI 0.5×, SGI 0.75×, SGI 1×, SGI 10×, SGI 20×, SGI 40×, SGI 80× and SGI 100× used. Ratios of fluorescence signals with and without Ag^+^ (Relative FL intensity, %) were recorded for comparison and are shown in Figure 2C. When SGI 1× was used (final concentration of 0.00625×), the relative FL intensity reached the biggest value of 3.93 ± 0.09, indicating the lowest signal-to-noise ratio. However, the difference value of FL intensity reached the maximum when DBR = 0.61, indicating the highest sensitivities. Therefore, the DBR value in the range of 0.06 to 0.61 seems appropriate for detection.

#### 3.3.3. Effect of Ionic Strength and pH for Ag^+^ Detection

SGI interacts with ssDNA through electrostatic interactions, while SGI binds to dsDNA through intercalation and minor groove binding. The groove binding and intercalation can be easily distinguished by changing the ionic strength and pH as previously reported [25]. With the increase of ionic strength, the groove-bound dyes will be released from the dsDNA, while it is difficult for the intercalated dyes. Some studies suggested that the primary interaction mechanism between SGI and dsDNA was minor groove binding [19,21]. Therefore, the ionic strength was optimized and controlled by adding NaNO_3_ due to Cl^−^ can form an insoluble product with Ag^+^. The effect of ionic strength on the Ag^+^ detection performance was investigated by varying the NaNO_3_ concentrations in the buffer solution under the fixed conditions of C_Ag_ = 10 μM, SSO = 1 μM, SGI = 1×, 100 mM MOPS and pH = 7.0. Moreover, the parallel experiments without Ag^+^ addition were conducted for the control. As shown in Figure 2D, upon addition of 500 mM NaNO_3_, the relative fluorescence intensity of SGI/SSO/Ag^+^ complex improved compared with the system with 100 mM NaNO_3_, 300 mM NaNO_3_, 700 mM NaNO_3,_ and 1000 mM NaNO_3_. Besides, the phenomenon of fluorescence blue shift was weakened. This phenomenon might be ascribed to the combination effects of ionic strength on the formation of C-Ag+-C mismatch and SGI staining [19,21]. Therefore, 500 mM NaNO_3_ was used throughout the experiments for Ag^+^ detection.

**Figure 3:**
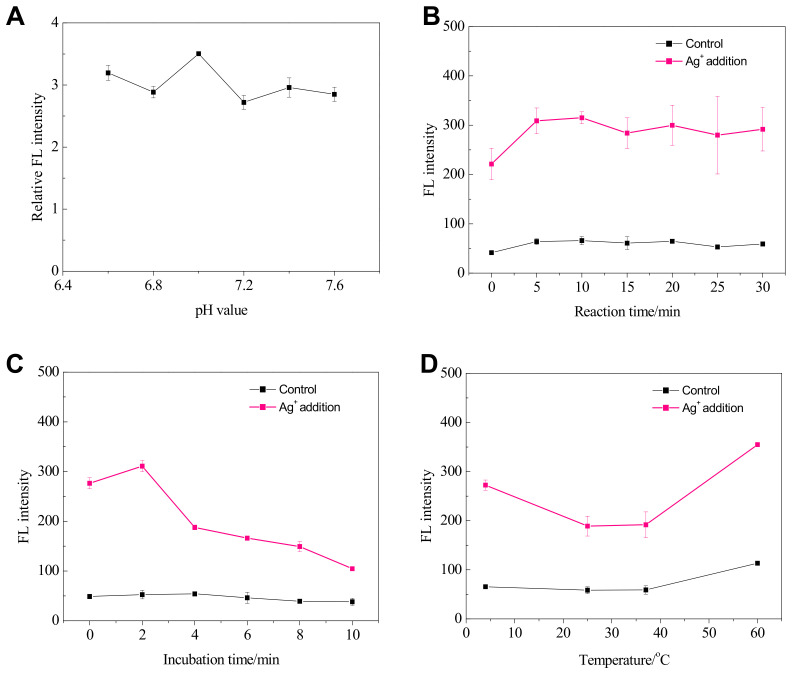
Effects of (**A**) pH value; (**B**) reaction time; (**C**) incubation time; and (**D**) temperature on fluorescence intensity for Ag^+^ detection (n = 2 if not specified).

The effect of pH values of buffer solution on the Ag^+^ detection performance was investigated under the fixed conditions of C_Ag_ = 10 μM, SGI = 1×, SSO = 1 μM, 100 mM MOPS buffer solution containing 500 mM NaNO_3_. Moreover, the parallel experiments without Ag^+^ addition were conducted for the control. Ratios of fluorescence signals with and without Ag^+^ (Relative FL intensity, %) were recorded for comparison and are shown in Figure 3A. The investigated pH values of MOPS buffer solution ranged from 6.6 to 7.6 had a slight impact on the detection performance. Relatively speaking, the pH = 7 shows the best performance; therefore, the buffer pH value of 7 was used in the following experiments.

#### 3.3.4. Effect of Reaction Time, Incubation Time and Temperature for Ag^+^ Detection

During the whole detection procedure, two steps were involved, including the reaction time for the C-Ag^+^-C formation and the subsequent incubation time for SGI staining. Both steps could possibly influence the performance [19,21,22,23]. The effect of reaction time for C-Ag^+^-C formation on the Ag^+^ detection performance was investigated under the fixed conditions of C_Ag_ = 10 μM, SGI = 1×, SSO = 1 μM, 100 mM MOPS buffer solution containing 500 mM NaNO_3,_ and pH = 7. Moreover, parallel experiments without Ag^+^ addition were conducted for the control. The fluorescence signals with and without Ag^+^ were recorded at different reaction times and shown in Figure 3B. The investigated reaction time had a slight impact on the detection performance once it was over 5 min. However, more than 10 min’s reaction time could increase the instability of FL intensities; therefore, 10 min was used in the following experiments for Ag^+^ detection. There is room for shortening the reaction time to 5 min.

The effect of incubation time for SGI staining on the Ag^+^ detection performance was investigated under the fixed conditions of C_Ag_ = 10 μM, SGI = 1×, SSO = 1 μM, 100 mM MOPS buffer solution containing 500 mM NaNO_3,_ and pH = 7. Moreover, the parallel experiments without Ag^+^ addition were conducted for the control. The fluorescence signals with and without Ag^+^ were recorded at different incubation times and are shown in Figure 3C. The investigated incubation time had a significant impact on detection performance. Once it was over 2 min, the FL intensity for Ag^+^ analysis decrease sharply; therefore, 2 min was used throughout the following experiments for Ag^+^ analysis.

Temperature is an essential factor in assessing the application prospect of in-field biosensing technology [26]; therefore, the effect of temperature on the Ag^+^ detection performance was investigated under the fixed conditions of C_Ag_ = 10 μM, SGI = 1×, SSO = 1 μM, 100 mM MOPS buffer solution containing 500 mM NaNO_3,_ and pH = 7. Moreover, parallel experiments without Ag^+^ addition were conducted for the control. It should be noted that the measuring procedures, including the reaction and incubation processes, were kept at the predefined temperatures. Due to the lack of a thermostat for the F-7000 fluorescence spectrometer, the fluorescence measurement was conducted at room temperature. Because the process was rapid, we thought the impact of temperature in the fluorescence signal acquirement was negligible. Figure 3D shows the fluorescence signals with and without Ag^+^ were recorded at different temperatures. The relative FL intensities remained stable in the temperature range from 25 °C to 37 °C. At the low temperature of 4 °C, the system showed the highest sensitivities and lowest signal-to-noise ratio. Also, at the temperature up to 60 °C, the FL intensity towards Ag^+^ addition still existed, although the background of fluorescence for the control also increased over 100 au.

### 3.4. Detection of Ag^+^

**Figure 4:**
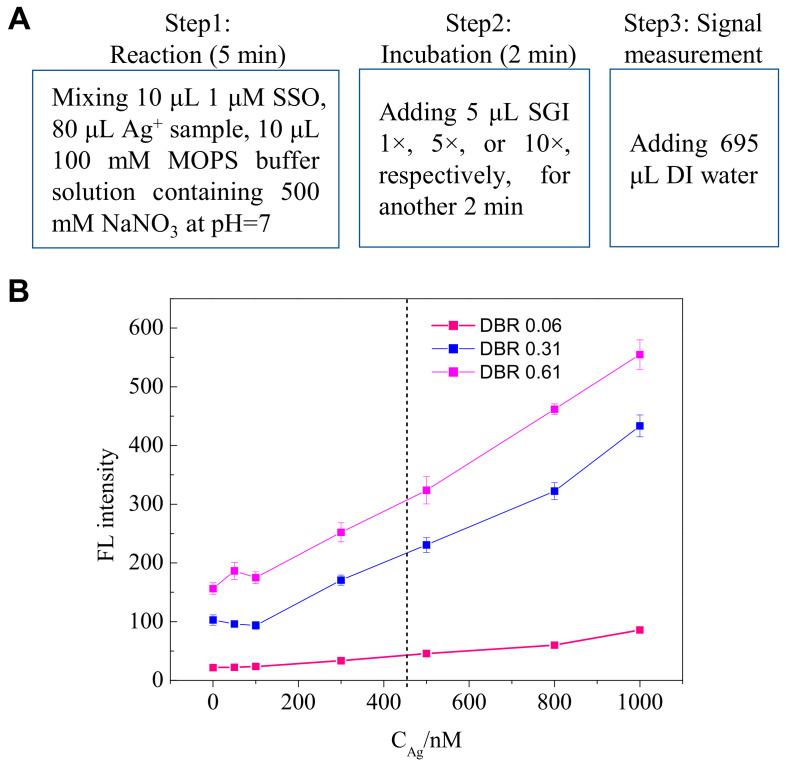
Detection performance of Ag^+^ by using this method. (**A**) Schematic diagram of optimized detection conditions; and (**B**) Fluorescence intensities for the detection of Ag^+^ from 0–1000 nM at different DBR values.

Under the optimized experimental conditions (Figure 4A: untreated ssDNA probe; 10 μL 1 μM SSO + 80 μL Ag^+^ standard samples + 10 μL MOPS buffer containing 500 mM NaNO_3_ (Ph = 7.0); 5 min for the formation of C-Ag^+^-C; 5 μL SGI (1×; 5×; 10×); 2 min for SGI staining; adding 695 μL DI water before fluorescence measurement; room temperature), Figure 4B shows the fluorescence intensities of the standard samples containing different concentrations of Ag^+^ at different DBR values, where the dashed line shows the silver limit in drinking water standard in China. The detection limits and linear relationships between fluorescence intensity and the concentration of Ag^+^ were summarized in Table 1.

The lower DBR values resulted in a lower signal-to-noise, therefore, a lower detection limit; however, weakening the assay’s sensitivities, which can be indicated by the slope of the regression line. Therefore, a balance between the detection limit and sensitivity is needed to be considered when choosing DBR values. We also investigated the repeatability of this method for Ag^+^ detection at three DBR values. The detection conditions are C_Ag_ = 400 nM, SSO = 1 μM, 100 mM MOPS buffer solution containing 500 mM NaNO_3_ and pH = 7.0. The reaction time for C-Ag^+^-C formation was 5 min and the incubation time for SGI staining was 2 min.

**Figure 5:**
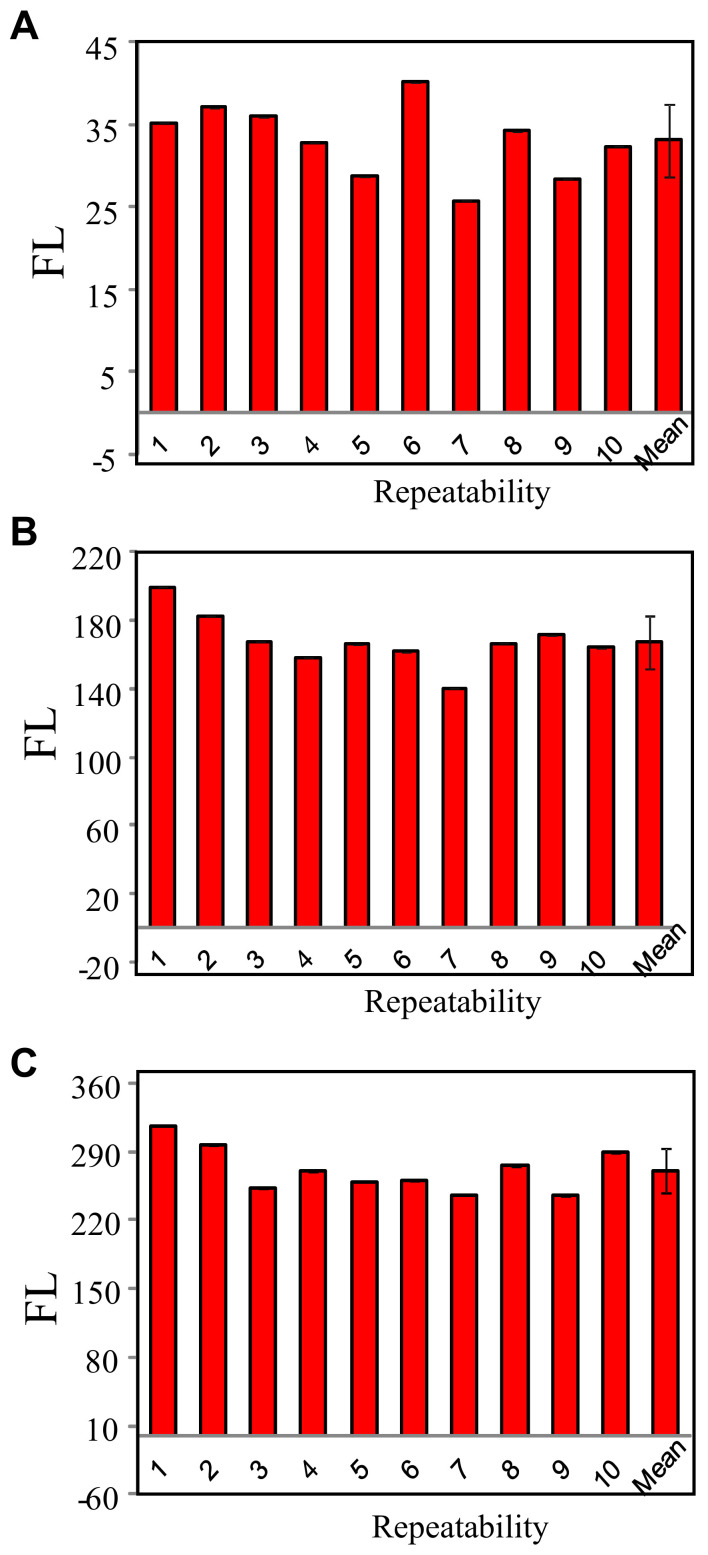
Repeatability of this bioassay for Ag^+^ analysis at different DBR values of (**A**) 0.06; (**B**) 0.31; and (**C**) 0.61.

As shown in Figure 5, the relative SD was 13.2%, 9.2%, and 8.6% for DBR values of 0.06, 0.31, 0.61, respectively, in ten times of measurements. The lower DBR value resulted in a lower detection limit. However, the limit for silver ion in all kinds of water standards is relatively high compared to this method’s detection limits under the three DBR values. Much attention should be paid to signal stability and sensitivity; therefore, we suggested using the DBR value of 0.61 in the following experiment (i.e., SGI 10×).

### 3.5. Selectivity

The specificity of the present system for Ag^+^ detection was evaluated by investigating the effects of common interference factors in the aqueous matrix. The interference factors include 11 items, as numbered in Table 2. The potential highest concentrations are mostly taken from the limits of V type water set by the Chinese environmental quality standard for surface water (GB3838-2002). In order to avoid precipitation, the anions used in most reagents are nitrates, such as copper nitrate, zinc nitrate, and so on.

**Figure 6:**
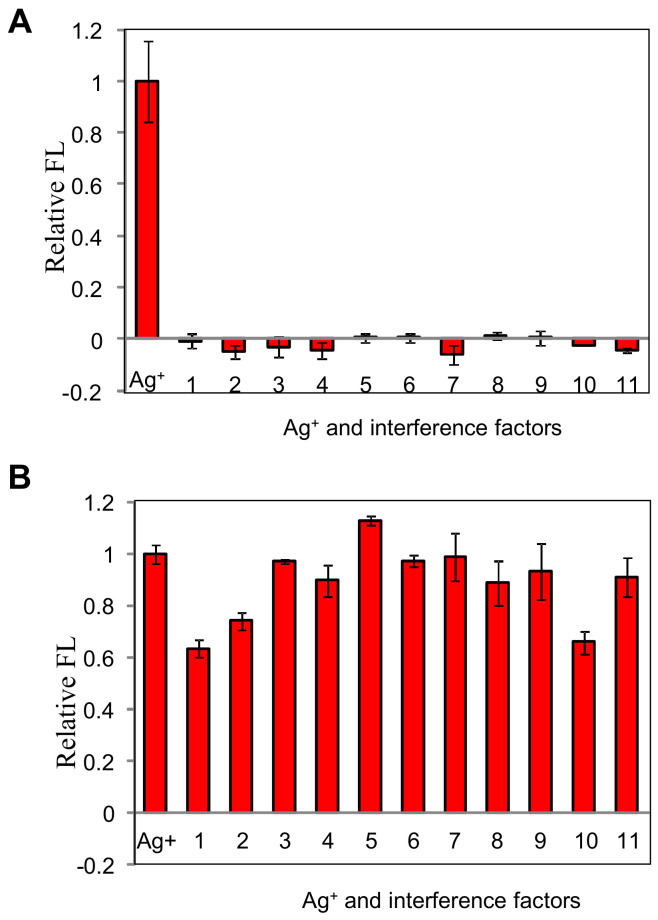
Selectivity of the proposed method against different interference factors (**A**) Ag^+^ and interference factors without Ag^+^, (**B**) Ag^+^ and interference factors with Ag^+^.

Figure 6A shows the peak signals at the silver concentrations of 700 nM. The interference factors at the upper limit concentrations are listed in Table 2. The relative fluorescence intensities caused by the interference factors compared with the target ion were below 5%, which were negligible. When the interference factors were added into the target ion solution, respectively, as shown in Figure 6B, no blatant interferences were observed in most cases; the relative errors were less than 10%.

However, three false-negative factors were confirmed, including the calcium hardness, magnesium hardness, and hypochlorite, which result in more than 20% relative errors compared with the response value towards the target. We attributed the big relative errors to two reasons: (1) the instability of silver hypochlorite formed by silver ions and hypochlorite; (2) the oxidation of hypochlorite, which would cause an unstable DNA probe. As the calcium and magnesium hardness increase, the ionic strength also increases, which might explain the decreased fluorescent signals in the presence of the calcium and magnesium hardness as shown in Figure 2D.

**Figure 7:**
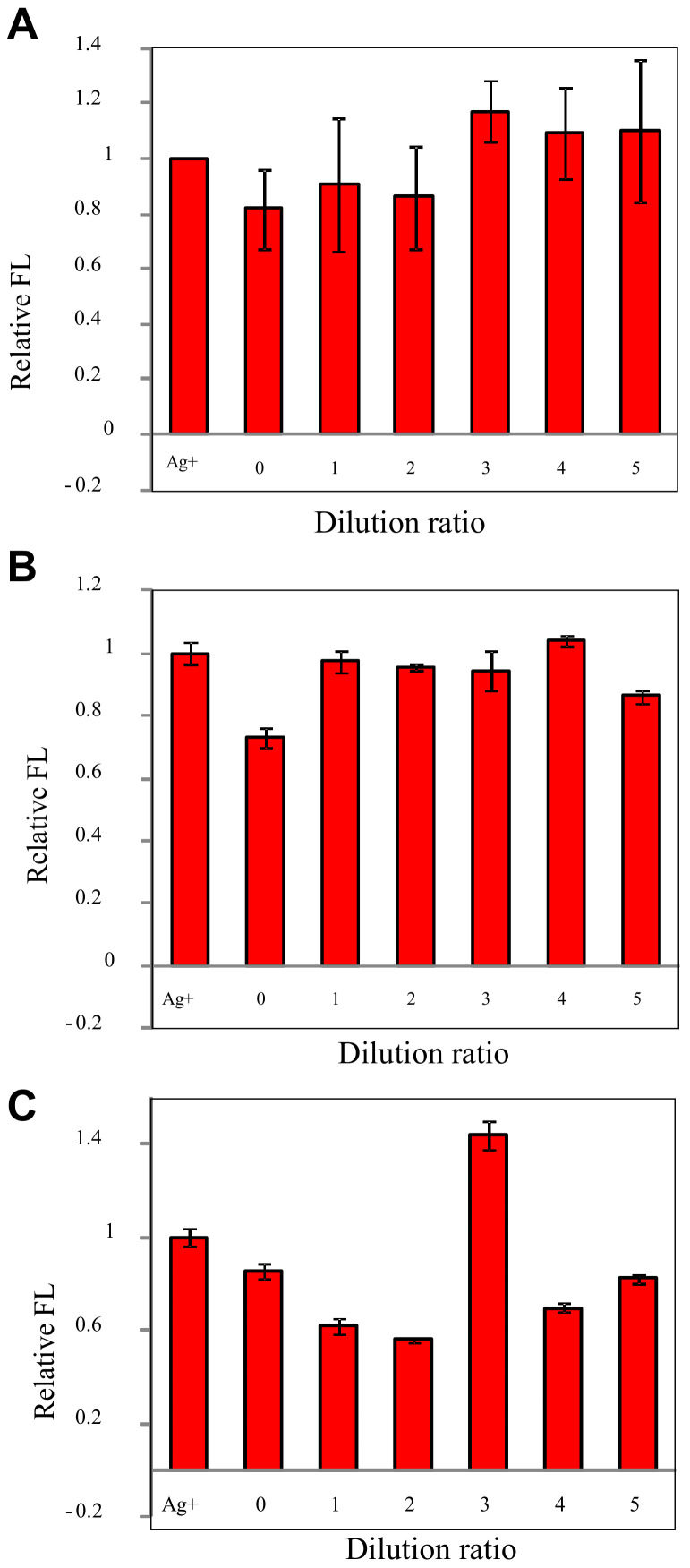
Tolerance level of the proposed method for different interferences factors: (**A**) calcium hardness, (**B**) magnesium hardness, and (**C**) hypochlorite.

Considering the high hardness in the ground water, the tolerance level of the calcium hardness method and magnesium hardness method by diluting the interference ions was investigated. As shown in Figure 7, the dilution ratio of over two can meet the request of making the relative error of less than 20%. Therefore, the tolerance levels were 200 mg/L for calcium ion and 120 mg/L for magnesium ion, respectively. However, the unstable false effect, maybe negative or positive, can be observed for the hypochlorite even if the interference ion was diluted five times. Therefore, more evaluations are needed if this method is used to detect tap or drinking water treated with the disinfection process.

### 3.6. Recovery

Two surface water (Danjiangkou Reservoir and Hetang lake in Tsinghua campus) and one groundwater type were tested for the recovery experiment’s real water matrix. A few pretreatments were added before the measurement, including boiling the water samples to precipitate the carbonate hardness, cooling the water to room temperature and filtering them through a filter with 0.22 μm nylon membrane, and then diluting five times using DI water before the recovery experiments.

Three levels of silver concentrations, including lower than, equal to, and higher than the silver limit in drinking water standard in China. Table 3 shows the recovery results in our experiment. The recovery abilities are satisfactory towards three types of diluted water. The recovery ratios were within the range of 79~110%.

## 4. Conclusions

We present an optimized detection procedure for Ag^+^ based on SGI and SSO. The optimized detection procedure includes three significant steps: (1) mixing 10 μL 1 μM SSO, 80 μL Ag^+^ sample, 10 μL 100 mM MOPS buffer solution containing 500 mM NaNO_3_ at pH = 7 for 5 min; (2) adding 5 μL SGI 10× for another 2 min; (3) adding 695 μL DI water for fluorescence measurement.

Under these conditions, the linear range of this method ranged from 100 nM to 1000 nM. Moreover, the detection limit is 59.9 nM. The total time for one assay is below 10 min; R.S.D. for ten measurements is 8.6%. In extreme cases, three false-negative factors were observed, including calcium hardness, magnesium hardness, and hypochlorite. This method’s recoveries towards three types of diluted real water are in the range of 79–110%.

## Figures and Tables

**Table 1:** Detection performance of the proposed method for Ag^+^ detection at different DBR values.

Performance	DBR = 0.06	DBR = 0.31	DBR = 0.61
Regression	FL = 0.053C_Ag_ + 18.36	FL = 0.32C_Ag_+67.13	FL = 0.423C_Ag_+125.3
Linear range	80–800 nM	100–800 nM	100–1000 nM
R^2^	0.998	0.997	0.997
Detection limit (3σ, n = 11)	6.4 nM	36.7 nM	59.9 nM

**Table 2:** Common interference factors existed in aqueous matrix.

Item	Interference	Potential Highest Concentrations are Chosen as the Upper Limits
**1**	Calcium Hardness	1000 mg/L as calcium carbonate, 400 mg/L as calcium (10 mM in Ca(NO_3_)_2_)
**2**	Magnesium Hardness	1000 mg/L as calcium carbonate, 240 mg/L as mgnesium (10 mM in Mg(NO_3_)_2_)
**3**	Copper	1.0 mg/L Cu^2+^ in Cu(NO_3_)_2_
**4**	Nitrate	10.0 mg/L NO_3_^-^ in NaNO_3_
**5**	Total iron	1.0 mg/L total Fe element in Fe(NO_3_)_3_
**6**	Zinc	2.0 mg/L Zn^2+^ in Zn(NO_3_)_2_
**7**	Fluoride	1.5 mg/L F^-^ in HF
**8**	Aluminum	0.2 mg/L Al^3+^ in Al(NO_3_)_3_
**9**	Manganese	0.2 mg/L Mn^2+^ in Mn(NO_3_)_2_
**10**	Hypochlorite	4.0 mg/L ClO^-^ in NaClO
**11**	Ammonium nitrogen	15 mg/L NH_4_^+^-N in NH_4_NO_3_

**Table 3:** Recoveries of this method in real water samples.

	Added nM	Measured nM	Recoveries %
Danjiangkou Reservoir	300	283	94.5 ± 5.6
	464	501	108 ± 36
	700	662	94.5 ± 3.3
Hetang Lake	300	253	84.3 ± 21
	464	368	79.3 ± 7.3
	700	572	81.7 ± 4.1
Groundwater in Tsinghua Yuan	300	287	95.6 ± 5.0
	464	387	83.3 ± 15
	700	638	91.1 ± 7.0
